# Supplementary material for: The effect of gender and parenting daughters on judgments of morally controversial companies
Source: PLoS One. 2021 Dec 1;16(12):e0260503. doi: 10.1371/journal.pone.0260503 (PMC8635371; doi:10.1371/journal.pone.0260503)
Supplement: S1 Appendix — (PDF) [file pone.0260503.s001.pdf]

S1 Appendix. Experimental Instructions

Industries
(previously used in: [1,2]**)**

Sin stocks

Abortion/Abortifacients. Companies owning or operating facilities where abortions are performed, abortion providers, abortifacient manufacturers.

Adult Entertainment. Companies targeted at the production or distribution of sexually explicit products and services, i.e., X-rated films, online products, production studios, printed materials, TV or radio programs, and adult clubs or bars.

Animal Testing*. Companies that do research or perform tests on animals for medical and cosmetic reasons (to determine safety and efficacy of particular products).

Controversial Weapons*. Companies involved in nuclear, biological, chemical weapons, cluster munitions, and antipersonnel mines.

Fur industry*. Companies that manufacture, sell, or distribute fur products.

Gambling*. Companies that manufacture, own, or operate gambling machines or equipment, casinos, lotteries and betting activities.

Tobacco*. Companies involved in the production, processing and wholesale distribution of tobacco products.

Conventional stocks

Air Freight/Logistics. Companies providing air freight transportation, air courier, and air logistics services.

Construction/Engineering. Companies engaged primarily in non-residential construction, as well as civil engineering companies.

Household Durables. Companies that manufacture consumer electronics, household appliances, houseware etc.

Marine. Companies providing maritime transportation of passengers or goods.

Road/Rail. Companies providing railroad and trucking transportation of passengers or goods.

Semiconductors/Semiconductor Equipment. Companies that manufacture semiconductors or semiconductor equipment.

Water Utilities. Companies that distribute water to consumers, also by being involved in water treatment.

* Five industries that were rated in Study 2.

After reading a description of the study and agreeing to participate, participants saw the following screens.

Screen 1 [Study 1]: Moral appropriateness of investing in morally controversial industries

Would it be morally appropriate if you invested in companies from the following industries? Please rate on a scale of 1 (*not at all*) to 7 (*completely*).

[Industries presented in random order; see “Industries”]

Screen 1 [Study 2]: Willingness to work in morally controversial industries

Would you be willing to quit a job operating in a conventional industry (e.g., in companies making household durables, semiconductors, or a water utility company) and start working in a company from

one of the following industries? Imagine that each of the industries listed below pays a wage that is 25% higher than what you would receive in more conventional industries. Please rate on a scale of 1 (not at all) to 7 (completely). Remember, there are no right or wrong choices, we only want to learn your opinions on this issue. So answer as honestly as you can.

[Industries presented in random order; see "Industries" (*)]

Screen 2: Attention check

Which of these industries was not mentioned during the survey?

[5 industries presented in random order]

Screen 3: Demographics (part 1)

What is your age (in years)?

What is your biological sex? [Male/female]

How many children do you have? This includes all biological and adopted children, as well as cases where you are step-parent or foster-parent.

Screen 4: Children

What is the age of your children (in years)?

Please select the biological sex of each of your children and select if they are your biological children, step children, foster children or adopted children.

Optional [conditional on earlier selections]

For your non-biological children, please state how old they were (in years), when you started being their parent (i.e., adopted them, became their step-parent, or became their foster-parent).

Given that your oldest children have the same age, please state what is the sex of your first-born child:

Screen 5: Demographics (part 2)

What is your marital status? [Single/married/divorced or widowed]

How many people are in your household?

What is the income in your household? [Less than $10,000/$10,000-$14,999/$15,000-$24,999/$25,000-$39,999/$40,000-$59,999/$60,000-$74,999/$75,000-$100,000/More than $100,000]

What is your highest achieved level of education? [Primary education/Secondary education/Bachelor or equivalent/Master or equivalent/Doctoral or equivalent]

What is your employment status? [Unemployed/Employee/Self-employed]

Self-rated investment knowledge item

To what extent do you disagree or agree with the following statement below? Please rate on a scale of 1 (*fully disagree*) to 7 (*fully agree*):

My investment knowledge is good.

Risk tolerance item [3]

How do you see yourself: are you generally a person who is fully prepared to take risks or do you try to avoid taking risks? Choose the appropriate number on a scale of 0 to 10, where: 0 = *not at all willing to take risks*, 10 = *very willing to take risks*.

Screen 6: Objective investment knowledge

(previously used in: [1,2]**)**

Investment knowledge test items. Items 1-4 were originally used in ref. [4]. Items 5-6 were originally used in ref. [5]. The items were presented in random order. The minimum score in the investment knowledge test was 0 and the maximum was 6.

1. Considering a long time period (e.g., 10 or 20 years), which asset normally gives the highest return: savings accounts, bonds or stocks?
2. Normally, which asset displays the highest fluctuations over time: savings accounts, bonds or stocks?
3. Stocks are normally riskier than bonds - is this statement True or False?
4. When an investor spreads money among different unrelated assets, does the risk of losing money: increase, decrease or stay the same?
5. If you were to invest $1000 in a stock fund, would it be possible to have less than $1000 when you decide to withdraw or move it to another fund?
6. High yield bond funds are invested in bonds with strong credit ratings - is this statement True or False?

Screen 7: Comments

If you have any comments, please put them in the box below [optional]

References

1. Niszczota P, Białek M. Women oppose sin stocks more than men do. Finance Research Letters. 2021;41: 101803. doi:10.1016/j.frl.2020.101803

2. Niszczota P, Kaszás D. Robo-investment aversion. PLOS ONE. 2020;15: e0239277. doi:10.1371/journal.pone.0239277

3. Dohmen T, Falk A, Huffman D, Sunde U. The Intergenerational Transmission of Risk and Trust Attitudes. Rev Econ Stud. 2012;79: 645–677. doi:10.1093/restud/rdr027

4. van Rooij M, Lusardi A, Alessie R. Financial literacy and stock market participation. J Financ Econ. 2011;101: 449–472. doi:10.1016/j.jfineco.2011.03.006

5. Agnew JR, Szykman LR. Asset Allocation and Information Overload: The Influence of Information Display, Asset Choice, and Investor Experience. J Behav Financ. 2005;6: 57–70. doi:10.1207/s15427579jpfm0602_2
