# Supplementary material for: The effect of gender and parenting daughters on judgments of morally controversial companies
Source: PLoS One. 2021 Dec 1;16(12):e0260503. doi: 10.1371/journal.pone.0260503 (PMC8635371; doi:10.1371/journal.pone.0260503)
Supplement: S1 Table — (PDF) [file pone.0260503.s002.pdf]

S1 Table. Summary statistics

	**All**<br>*N* = 1,416	**Men**<br>*N* = 766	**Women**<br>*N* = 650	***Men vs women***
**Age**	38.6 (12.1)	38.2 (12.0)	39.2 (12.2)	
**Number of children**	0.92 (1.21)	0.75 (1.08)	1.12 (1.32)	***
**Number of daughters**	0.44 (0.75)	0.33 (0.62)	0.57 (0.85)	***
**Number of sons**	0.48 (0.80)	0.43 (0.78)	0.55 (0.82)	***
**Household size**	2.75 (1.37)	2.61 (1.35)	2.91 (1.38)	***
**Household income**				
Under $10,000	49 (3.46%)	23 (3.00%)	26 (4.00%)	
$10,000-15,000	54 (3.81%)	33 (4.31%)	21 (3.23%)	
$15,000-25,000	107 (7.56%)	56 (7.31%)	51 (7.85%)	
$25,000-40,000	240 (16.9%)	135 (17.6%)	105 (16.2%)	
$40,000-60,000	307 (21.7%)	178 (23.2%)	129 (19.8%)	
$60,000-40,000	214 (15.1%)	107 (14.0%)	107 (16.5%)	
$75,000-100,000	225 (15.9%)	111 (14.5%)	114 (17.5%)	
Over $100,000	220 (15.5%)	123 (16.1%)	97 (14.9%)	
**Education**				***
Primary	55 (3.88%)	33 (4.31%)	22 (3.38%)	
Secondary	408 (28.8%)	206 (26.9%)	202 (31.1%)	
Bachelor or equivalent	748 (52.8%)	430 (56.1%)	318 (48.9%)	
Masters or equivalent	181 (12.8%)	80 (10.4%)	101 (15.5%)	
PhD or equivalent	24 (1.69%)	17 (2.22%)	7 (1.08%)	
**Marital status**				***
Single	637 (45.0%)	392 (51.2%)	245 (37.7%)	
Married	662 (46.8%)	329 (43.0%)	333 (51.2%)	
Divorced or widowed	117 (8.26%)	45 (5.87%)	72 (11.1%)	
**Employment status**				
Employee	971 (68.6%)	566 (73.9%)	405 (62.3%)	
Self-employed	225 (15.9%)	110 (14.4%)	115 (17.7%)	
Unemployed	220 (15.5%)	90 (11.7%)	130 (20.0%)	
**Risk tolerance**	4.47 (2.52)	4.83 (2.41)	4.05 (2.57)	***
**Subjective investment knowledge**	3.95 (1.72)	4.26 (1.62)	3.58 (1.76)	***
**Objective investment knowledge**	4.41 (1.41)	4.56 (1.43)	4.23 (1.37)	***

Notes: Computed on pooled dataset (i.e., containing Study 1 and Study 2 samples). The statistics consist of means (and standard deviations) for numerical data, and the number of participants in each group for categorical data.

*** $p < 0.01$ in *t*-test.
