# Supplementary material for: The effect of gender and parenting daughters on judgments of morally controversial companies
Source: PLoS One. 2021 Dec 1;16(12):e0260503. doi: 10.1371/journal.pone.0260503 (PMC8635371; doi:10.1371/journal.pone.0260503)
Supplement: S2 Table — (PDF) [file pone.0260503.s003.pdf]

S2 Table. The effect of the number of daughters

	Investment		Employment	
	All	*Biological*	*All*	*Biological*
Gender (0 = *m*, 1 = *f*)	–0.51 *** (0.16)	–0.47 *** (0.16)	–0.27 * (0.14)	–0.25 * (0.14)
Daughters > 0	–0.36 (0.23)	–0.33 (0.25)	–0.02 (0.23)	–0.00 (0.24)
Numbers of daughters after first	–0.42 (0.34)	–0.37 (0.40)	–0.30 (0.23)	–0.20 (0.35)
Sons > 0	0.26 (0.24)	0.26 (0.25)	–0.14 (0.20)	–0.11 (0.21)
Numbers of sons after first	–0.19 (0.20)	–0.23 (0.23)	0.19 (0.18)	0.13 (0.23)
Gender × [Daughters > 0]	0.63 ** (0.30)	0.55 * (0.31)	–0.05 (0.28)	–0.10 (0.30)
Gender × [Numbers of daughters after first]	0.16 (0.41)	0.27 (0.46)	0.07 (0.29)	–0.03 (0.40)
Gender × [Sons > 0]	–0.05 (0.29)	–0.15 (0.31)	0.19 (0.26)	0.15 (0.27)
Gender × [Numbers of sons after first]	–0.14 (0.29)	–0.15 (0.42)	–0.30 (0.22)	–0.23 (0.28)
Risk tolerance	0.17 *** (0.03)	0.16 *** (0.03)	0.12 *** (0.03)	0.13 *** (0.03)
Objective investment knowledge	-0.29 *** (0.04)	-0.27 *** (0.05)	-0.11 *** (0.04)	-0.09 ** (0.04)
Subjective investment knowledge	0.08 (0.05)	0.07 (0.05)	0.17 *** (0.04)	0.16 *** (0.04)
Marital status: married	0.25 (0.18)	0.25 (0.18)	0.40 *** (0.15)	0.40 ** (0.16)
Marital status: divorced or widowed	0.23 (0.26)	0.20 (0.26)	-0.22 (0.20)	-0.19 (0.21)
Education: doctoral level or equivalent	0.54 (0.50)	0.60 (0.57)	-0.71 (0.47)	-0.92 ** (0.45)
Education: Master's degree or equivalent	0.47 *** (0.18)	0.43 ** (0.18)	-0.11 (0.17)	-0.11 (0.17)
Education: primary school	-0.38 (0.39)	-0.35 (0.38)	0.21 (0.26)	0.21 (0.28)
Education: secondary school	-0.32 ** (0.15)	-0.30 * (0.16)	0.11 (0.12)	0.08 (0.13)
Employment: self-employed	0.16 (0.17)	0.15 (0.18)	-0.65 *** (0.15)	-0.62 *** (0.16)
Employment: unemployed	-0.22 (0.20)	-0.24 (0.20)	-0.46 *** (0.14)	-0.48 *** (0.14)
Age (logged)	-1.03 *** (0.27)	-0.97 *** (0.29)	0.00 (0.20)	-0.04 (0.20)
Household income (midpoint, logged)	-0.15 (0.10)	-0.11 (0.10)	-0.33 *** (0.09)	-0.32 *** (0.09)
N	634	604	781	742
Adjusted R^2	0.256	0.232	0.185	0.185
Daughters vs sons (for men)	$p = 0.22$	$p = 0.34$	$p = 0.08$	$p = 0.11$

Notes: Robust standard errors are in parentheses. *** $p < 0.01$ ** $p < 0.05$ * $p < 0.1$
