# Supplementary material for: The effect of gender and parenting daughters on judgments of morally controversial companies
Source: PLoS One. 2021 Dec 1;16(12):e0260503. doi: 10.1371/journal.pone.0260503 (PMC8635371; doi:10.1371/journal.pone.0260503)
Supplement: S3 Table — (PDF) [file pone.0260503.s004.pdf]

S3 Table. Analysis of male and female subsamples: a comparison of the effect of having daughters, and the number of daughters conditional on the number of children

	**Men**		**Women**	
	Investment	*Employment*	*Investment*	*Employment*
Panel A. Dummy variable only [6]				
Daughters > 0	–0.54 **<br>(0.24)	–0.20<br>(0.22)	0.19<br>(0.19)	–0.21<br>(0.18)
Risk tolerance	0.18 ***<br>(0.04)	0.13 ***<br>(0.03)	0.14 ***<br>(0.04)	0.10 ***<br>(0.04)
Objective investment knowledge	-0.25 ***<br>(0.06)	-0.08<br>(0.05)	-0.36 ***<br>(0.06)	-0.15 **<br>(0.06)
Subjective investment knowledge	0.08<br>(0.07)	0.16 ***<br>(0.05)	0.13 **<br>(0.06)	0.19 ***<br>(0.05)
Marital status: married	0.51 **<br>(0.25)	0.25<br>(0.21)	0.18<br>(0.21)	0.57 ***<br>(0.19)
Marital status: divorced or widowed	0.22<br>(0.42)	-0.04<br>(0.33)	0.39<br>(0.33)	-0.24<br>(0.24)
Education: doctoral level or equivalent	0.85<br>(0.69)	-0.80<br>(0.51)	0.10<br>(0.82)	-0.74<br>(2.28)
Education: Master’s degree or equivalent	0.64 **<br>(0.26)	-0.35<br>(0.24)	0.27<br>(0.25)	0.07<br>(0.23)
Education: primary school	-0.52<br>(0.46)	0.42<br>(0.40)	0.18<br>(0.81)	-0.03<br>(0.31)
Education: secondary school	-0.08<br>(0.23)	-0.04<br>(0.18)	-0.61 ***<br>(0.20)	0.27<br>(0.17)
Employment: self-employed	0.43<br>(0.27)	-0.88 ***<br>(0.23)	-0.11<br>(0.22)	-0.38 *<br>(0.21)
Employment: unemployed	0.18<br>(0.33)	-0.32<br>(0.23)	-0.51 **<br>(0.24)	-0.52 ***<br>(0.17)
Age (logged)	-1.11 **<br>(0.44)	0.22<br>(0.29)	-1.08 ***<br>(0.34)	-0.28<br>(0.29)
Household income (midpoint, logged)	-0.02<br>(0.17)	-0.31 ***<br>(0.12)	-0.24 *<br>(0.13)	-0.38 ***<br>(0.14)
Observations	344	422	290	359
Adjusted R^2	0.181	0.132	0.340	0.202
Panel B. Number of daughters and number of children (Washington, 2008; Van Effenterre, 2020)				
Number of daughters	–0.42 **<br>(0.20)	–0.17<br>(0.19)	0.10<br>(0.20)	–0.12<br>(0.12)
Number of children	–0.02<br>(0.13)	–0.00<br>(0.13)	–0.06<br>(0.13)	–0.05<br>(0.08)
Risk tolerance	0.18 ***<br>(0.04)	0.13 ***<br>(0.04)	0.14 ***<br>(0.04)	0.10 ***<br>(0.04)
Objective investment knowledge	-0.25 ***<br>(0.06)	-0.07<br>(0.06)	-0.38 ***<br>(0.07)	-0.15 **<br>(0.06)
Subjective investment knowledge	0.07<br>(0.07)	0.16 ***<br>(0.05)	0.14 **<br>(0.06)	0.19 ***<br>(0.05)

Marital status: married	0.54 ** (0.27)	0.24 (0.22)	0.26 (0.23)	0.62 *** (0.20)
Marital status: divorced or widowed	0.25 (0.46)	-0.03 (0.33)	0.45 (0.34)	-0.22 (0.24)
Education: doctoral level or equivalent	0.80 (0.67)	-0.79 (0.51)	0.13 (0.82)	-0.76 (2.22)
Education: Master’s degree or equivalent	0.64 ** (0.26)	-0.34 (0.24)	0.26 (0.25)	0.07 (0.23)
Education: primary school	-0.56 (0.46)	0.44 (0.40)	0.17 (0.79)	-0.04 (0.31)
Education: secondary school	-0.10 (0.23)	-0.04 (0.18)	-0.60 *** (0.20)	0.29 * (0.17)
Employment: self-employed	0.47 * (0.28)	-0.89 *** (0.23)	-0.11 (0.22)	-0.39 * (0.21)
Employment: unemployed	0.19 (0.33)	-0.37 (0.23)	-0.53 ** (0.24)	-0.50 *** (0.17)
Age (logged)	-1.09 ** (0.44)	0.24 (0.28)	-1.04 *** (0.35)	-0.23 (0.28)
Household income (midpoint, logged)	-0.01 (0.17)	-0.30 *** (0.12)	-0.24 * (0.13)	-0.36 ** (0.14)
Controls	Yes	Yes	Yes	Yes
Observations	344	421	290	359
Adjusted R^2	0.184	0.130	0.336	0.206

Notes: Robust standard errors are in parentheses. *** $p < 0.01$ ** $p < 0.05$ * $p < 0.1$
