# Supplementary material for: The effect of gender and parenting daughters on judgments of morally controversial companies
Source: PLoS One. 2021 Dec 1;16(12):e0260503. doi: 10.1371/journal.pone.0260503 (PMC8635371; doi:10.1371/journal.pone.0260503)
Supplement: S4 Table — (PDF) [file pone.0260503.s005.pdf]

**S4 Table. Test of female socialization hypothesis and effect of first-born child**

| | Investment | Employment |
|---|---|---|
| | *Biological* | *Biological* |
| ***Panel A. Female socialization*** | | |
| Gender (0 = *m*, 1 = *f*) | –0.45 *** (0.16) | –0.21 (0.14) |
| Years of parenting daughters (logged) | –0.46 (0.30) | –0.01 (0.28) |
| Years of parenting sons (logged) | 0.02 (0.29) | –0.22 (0.27) |
| Gender × [years of parenting daughters (logged)] | 0.72 * (0.38) | –0.34 (0.31) |
| Gender × [years of parenting sons (logged)] | –0.16 (0.36) | 0.10 (0.32) |
| Risk tolerance | 0.17 *** (0.03) | 0.13 *** (0.03) |
| Objective investment knowledge | -0.28 *** (0.05) | -0.10 ** (0.04) |
| Subjective investment knowledge | 0.08 * (0.05) | 0.16 *** (0.04) |
| Marital status: married | 0.30 * (0.17) | 0.43 *** (0.15) |
| Marital status: divorced or widowed | 0.22 (0.26) | -0.14 (0.20) |
| Education: doctoral level or equivalent | 0.64 (0.56) | -0.91 ** (0.44) |
| Education: Master's degree or equivalent | 0.40 ** (0.18) | -0.11 (0.17) |
| Education: primary school | -0.33 (0.38) | 0.24 (0.27) |
| Education: secondary school | -0.31 ** (0.16) | 0.10 (0.13) |
| Employment: self-employed | 0.13 (0.18) | -0.62 *** (0.16) |
| Employment: unemployed | -0.26 (0.20) | -0.48 *** (0.14) |
| Age (logged) | -0.98 *** (0.29) | 0.01 (0.21) |
| Household income (midpoint, logged) | -0.13 (0.10) | -0.32 *** (0.09) |
| Observations | 604 | 742 |
| Adjusted $R^2$ | 0.230 | 0.189 |
| *Panel B. Subsample analysis comparing against solely against participants with one child* | | |
| Gender (0 = *m*, 1 = *f*) | –0.51 *** (0.17) | –0.29 * (0.15) |
| Daughters > 0 | –0.41 (0.36) | 0.13 (0.39) |
| Sons > 0 | 0.35 (0.30) | 0.24 (0.30) |
| Gender × [Daughters > 0] | 0.78 * (0.43) | 0.26 (0.46) |

| | | |
|---|---|---|
| Gender × [Sons > 0] | –0.24<br>(0.40) | –0.05<br>(0.37) |
| Risk tolerance | 0.18 ***<br>(0.03) | 0.12 ***<br>(0.03) |
| Objective investment knowledge | -0.24 ***<br>(0.05) | -0.10 *<br>(0.05) |
| Subjective investment knowledge | 0.06<br>(0.06) | 0.20 ***<br>(0.05) |
| Marital status: married | 0.45 **<br>(0.20) | 0.34 *<br>(0.18) |
| Marital status: divorced or widowed | 0.43<br>(0.33) | -0.18<br>(0.28) |
| Education: doctoral level or equivalent | 0.66<br>(0.69) | -0.58<br>(0.68) |
| Education: Master's degree or equivalent | 0.38 *<br>(0.22) | 0.07<br>(0.22) |
| Education: primary school | -0.26<br>(0.41) | 0.20<br>(0.32) |
| Education: secondary school | -0.18<br>(0.18) | 0.04<br>(0.15) |
| Employment: self-employed | 0.16<br>(0.21) | -0.57 ***<br>(0.18) |
| Employment: unemployed | -0.08<br>(0.23) | -0.46 **<br>(0.18) |
| Age (logged) | -1.29 ***<br>(0.32) | -0.02<br>(0.24) |
| Household income (midpoint, logged) | -0.14<br>(0.11) | -0.32 ***<br>(0.10) |
| Observations | 455 | 544 |
| Adjusted $R^2$ | 0.218 | 0.199 |

*Notes:* Robust standard errors are in parentheses. *** $p < 0.01$ ** $p < 0.05$ * $p < 0.1$
