# Supplementary material for: The effect of gender and parenting daughters on judgments of morally controversial companies
Source: PLoS One. 2021 Dec 1;16(12):e0260503. doi: 10.1371/journal.pone.0260503 (PMC8635371; doi:10.1371/journal.pone.0260503)
Supplement: S5 Table — (PDF) [file pone.0260503.s006.pdf]

S5 Table. Influence of differences in household income levels

	Investment		Employment	
	Low income	*High income*	*Low income*	*High income*
Gender (0 = *m*, 1 = *f*)	–0.48 * (0.27)	–0.50 ** (0.21)	–0.26 (0.21)	–0.28 (0.21)
Daughters > 0	–0.52 (0.33)	–0.48 (0.33)	0.10 (0.30)	–0.16 (0.30)
Sons > 0	–0.08 (0.34)	0.45 (0.29)	–0.38 (0.29)	0.26 (0.28)
Gender × [Daughters > 0]	0.63 (0.41)	0.53 (0.41)	–0.02 (0.35)	–0.25 (0.35)
Gender × [Sons > 0]	0.44 (0.40)	–0.73 * (0.38)	0.21 (0.34)	–0.12 (0.35)
Risk tolerance	0.20 *** (0.05)	0.15 *** (0.04)	0.15 *** (0.03)	0.09 ** (0.04)
Objective investment knowledge	-0.27 *** (0.07)	-0.29 *** (0.07)	-0.12 ** (0.06)	-0.08 (0.06)
Subjective investment knowledge	0.12 * (0.07)	0.06 (0.06)	0.23 *** (0.06)	0.12 ** (0.05)
Marital status: married	0.27 (0.31)	0.31 (0.22)	0.25 (0.23)	0.51 ** (0.22)
Marital status: divorced or widowed	-0.17 (0.39)	0.61 * (0.36)	-0.54 * (0.32)	0.12 (0.27)
Education: doctoral level or equivalent	0.47 (1.38)	0.59 (0.65)	-2.25 *** (0.54)	-0.09 (0.61)
Education: Master’s degree or equivalent	0.54 * (0.31)	0.42 * (0.22)	-0.50 * (0.28)	0.04 (0.21)
Education: primary school	-0.29 (0.43)	-0.30 (0.89)	0.30 (0.37)	0.30 (0.38)
Education: secondary school	-0.25 (0.23)	-0.41 ** (0.20)	0.09 (0.17)	0.22 (0.19)
Employment: self-employed	0.31 (0.26)	-0.07 (0.24)	-0.78 *** (0.23)	-0.53 ** (0.23)
Employment: unemployed	-0.17 (0.36)	-0.26 (0.24)	-0.56 *** (0.20)	-0.34 (0.21)
Age (logged)	-1.13 *** (0.41)	-1.18 *** (0.41)	0.12 (0.30)	-0.39 (0.30)
Household income (midpoint, logged)	-0.14 (0.16)	-0.04 (0.29)	-0.38 *** (0.12)	-0.45 ** (0.22)
Observations	299	332	371	399
Adjusted R^2	0.263	0.225	0.276	0.109

Notes: Robust standard errors are in parentheses. *** $p < 0.01$ ** $p < 0.05$ * $p < 0.1$
