# Supplementary material for: The effect of gender and parenting daughters on judgments of morally controversial companies
Source: PLoS One. 2021 Dec 1;16(12):e0260503. doi: 10.1371/journal.pone.0260503 (PMC8635371; doi:10.1371/journal.pone.0260503)
Supplement: S6 Table — (PDF) [file pone.0260503.s007.pdf]

S6 Table. Judgment of individual industries (Studies 1 and 2 pooled)

	Animal testing	Controversial weapons	Fur industry	Gambling	Tobacco
Gender (0 = *m*, 1 = *f*)	–0.41 *** (0.13)	–0.31 ** (0.14)	–0.36 ** (0.14)	–0.24 (0.15)	–0.27 * (0.14)
Daughters > 0	–0.41 ** (0.17)	–0.14 (0.17)	–0.27 (0.18)	–0.03 (0.19)	–0.05 (0.18)
Sons > 0	0.11 (0.17)	0.05 (0.17)	0.32 * (0.18)	–0.07 (0.19)	0.01 (0.18)
Gender × [Daughters > 0]	0.27 (0.22)	–0.09 (0.22)	0.22 (0.23)	0.22 (0.25)	0.11 (0.24)
Gender × [Sons > 0]	0.03 (0.22)	0.04 (0.22)	0.01 (0.23)	0.02 (0.24)	0.02 (0.23)
Risk tolerance	0.12 *** (0.02)	0.18 *** (0.02)	0.13 *** (0.02)	0.15 *** (0.02)	0.14 *** (0.02)
Objective investment knowledge	-0.18 *** (0.04)	-0.17 *** (0.04)	-0.21 *** (0.04)	0.05 (0.04)	-0.15 *** (0.04)
Subjective investment knowledge	0.22 *** (0.03)	0.18 *** (0.03)	0.22 *** (0.04)	0.12 *** (0.04)	0.18 *** (0.04)
Marital status: married	0.56 *** (0.14)	0.43 *** (0.14)	0.53 *** (0.14)	-0.24 (0.15)	0.34 ** (0.15)
Marital status: divorced or widowed	-0.17 (0.21)	-0.20 (0.21)	0.07 (0.22)	0.09 (0.24)	0.14 (0.23)
Education: doctoral level or equivalent	0.52 (0.38)	0.20 (0.38)	0.45 (0.40)	-0.23 (0.42)	-0.11 (0.40)
Education: Master's degree or equivalent	0.12 (0.15)	-0.05 (0.16)	0.17 (0.16)	-0.17 (0.17)	-0.03 (0.16)
Education: primary school	-0.39 (0.26)	0.47 * (0.26)	-0.14 (0.27)	0.47 (0.29)	0.24 (0.28)
Education: secondary school	-0.22 * (0.12)	-0.11 (0.12)	0.03 (0.12)	0.02 (0.13)	0.19 (0.13)
Employment: self-employed	-0.27 * (0.14)	-0.27 * (0.14)	-0.36 ** (0.15)	-0.35 ** (0.16)	-0.46 *** (0.15)
Employment: unemployed	-0.27 * (0.14)	-0.40 *** (0.15)	-0.41 *** (0.15)	-0.20 (0.16)	-0.46 *** (0.15)
Age (logged)	-0.28 (0.20)	-0.06 (0.20)	-0.28 (0.20)	0.47 ** (0.22)	-0.25 (0.21)
Household income (midpoint, logged)	-0.31 *** (0.08)	-0.25 *** (0.08)	-0.35 *** (0.08)	-0.07 (0.09)	-0.28 *** (0.08)
Adjusted R^2	1,415	1,415	1,415	1,415	1,415
R^2 adjusted	0.177	0.177	0.171	0.059	0.115

Notes: *** $p < 0.01$ ** $p < 0.05$ * $p < 0.1$
