# Supplementary material for: The effect of gender and parenting daughters on judgments of morally controversial companies
Source: PLoS One. 2021 Dec 1;16(12):e0260503. doi: 10.1371/journal.pone.0260503 (PMC8635371; doi:10.1371/journal.pone.0260503)
Supplement: Attachment — Submitted filename: PLOS_2021.pdf [file pone.0260503.s008.pdf]

The effect of gender and parenting daughters on judgments
of morally controversial companies

Referee Report

This paper studies the importance of gender of respondents and their children for their willingness to invest in and work for sinful companies. Using survey data on a sample of 1000 respondents, the paper finds that men with daughters are less likely to invest in sinful companies, but similar effect is absent for the decisions to work. In turn, females with daughters are less likely to both invest in and work for sinful companies. The paper interprets the findings in terms of their contribution to wage income gap.

The paper contributes to a literature relating social norms to financial outcomes. Research to date has shown the effect of social norms for aggregate samples as well as for gender-stratified samples. The contribution of this paper is to extend these results into a setting in which gender of a surveyed person interacts with a gender of his/her offspring. I find the two experiments presented in the paper quite interesting and the conclusions from the paper are quite worth reporting. I also think the paper is generally well written. I have a number of suggestions that I hope can improve the readability of the paper a bit more.

Specific Comments

1. My first problem reading the paper is that it omits some important information that would be useful to report when presenting the results. For example, the results from the regressions do not show the list of controls that are included and more so the coefficients of these variables. These would be useful as they would give a reader a better sense of the properties of the sample. Similarly, I did not find good evidence on the definition of

controversial sectors. Perhaps this has been reported in the past study, but I think it would be necessary to bring up some of these materials to the current draft as well.

2. Like the authors, I am also worried a bit about the Rabin effect, that is, whether intent to do something is equivalent to the act of doing it itself. Is there some evidence that the authors could bring up that this distinction is gender or gender/offspring indifferent? I think this would be a partial way to address the concern whether one can interpret the results as economically meaningful.
3. It would be helpful to clarify that the results from both experiments are extensive margin results, which do not allow for the possibility of the variation in intensity of action. This is particularly true for investment decisions. In some sense, the experiments assume the strict exclusionary screening on its participants.
4. It would be useful to provide some discussion of the economic magnitudes. Can you link your results to some form of aggregates? When you discuss issues like gender gap, how should we think of these ideas in the context of the fraction of jobs that are available in the controversial industries, average pay in these jobs, etc?
5. In the discussion section, the authors argue that their results suggest that men might internalize the preferences of their daughters. I find this result quite surprising. Does this mean the daughters are already grown-up individuals? If so, it would be useful to bring up some evidence on the demographics of these daughters/sons. My interpretation would be that having a daughter may carry a different emotional load and men may want to cater to this social norm pressure. Can the authors rule out such explanation? What is the basis for such inference?
