# Supplementary material for: The effect of gender and parenting daughters on judgments of morally controversial companies
Source: PLoS One. 2021 Dec 1;16(12):e0260503. doi: 10.1371/journal.pone.0260503 (PMC8635371; doi:10.1371/journal.pone.0260503)
Supplement: Attachment — Submitted filename: Response to Reviewers.pdf [file pone.0260503.s009.pdf]

Response to Reviewers

The effect of gender and parenting daughters on judgments of morally controversial companies

PONE-D-21-22149

Specific Comments

1. My first problem reading the paper is that it omits some important information that would be useful to report when presenting the results. For example, the results from the regressions do not show the list of controls that are included and more so the coefficients of these variables. These would be useful as they would give a reader a better sense of the properties of the sample. Similarly, I did not find good evidence on the definition of controversial sectors. Perhaps this has been reported in the past study, but I think it would be necessary to bring up some of these materials to the current draft as well.

> We'd like to thank the Reviewer for these suggestions. Our tables were indeed unorthodox, in that they did not list which control variables were used, and what were their estimates. We have amended this: now all our tables (including the supplementary tables) include estimates for all control variables.
>
> We now also mention in the text which controversial and conventional stocks we used, additionally pointing the reader to descriptions (in the Appendix). This should have been indicated in the earlier version of the paper – we appreciate that the Reviewer has pointed out this issue, as it enhances the readability of the manuscript.

2. Like the authors, I am also worried a bit about the Rabin effect, that is, whether intent to do something is equivalent to the act of doing it itself. Is there some evidence that the authors could bring up that this distinction is gender or gender/offspring indifferent? I think this would be a partial way to address the concern whether one can interpret the results as economically meaningful.

> This is an excellent observation. Indeed, the differences that we observed could potentially be the result of gender differences in hypothetical bias, i.e. the difference between the intent to act and actual action. However, there is no consistent evidence on whether this bias is different between men and women (Johansson-Stenman & Svedsäter, 2012): significant differences, if present, mostly show that the hypothetical bias is actually *lower* in women. This would suggest that women – relative to men – state preferences that are not that far away from real-life (revealed) preferences (in our case: a preference of *not investing* in morally controversial companies, and *not wanting* to be employed by them). (We are unaware of any studies that would investigate how the gender of children might impact hypothetical bias.)
>
> We address this issue in the Discussion.

3. It would be helpful to clarify that the results from both experiments are extensive margin results, which do not allow for the possibility of the variation in intensity of action. This is particularly true for investment decisions. In some sense, the experiments assume the strict exclusionary screening on its participants.

Another fine point. Indeed, we designed both studies with the intention of studying extensive margins. We now confirm that this is the case in the Introduction. We also point out that we assumed that participants would be able to treat our task as one that relates to extensive margins, and not intensive margins (the latter could be especially relevant if they actually invested or were employed in companies from the listed industries). Participants were assumed to be robust to such cases, indeed being able to correctly infer that we were after the extensive margin.

We now notice in the Discussion that this is an important distinction, and that studying intensive margins results could potentially lead to different results.

4. It would be useful to provide some discussion of the economic magnitudes. Can you link your results to some form of aggregates? When you discuss issues like gender gap, how should we think of these ideas in the context of the fraction of jobs that are available in the controversial industries, average pay in these jobs, etc?

This is a very useful comment. It is important to point out to the reader that this is actually a salient issue, given that the proportion of companies that could be considered as morally controversial is considerable. We provide a (reasonable, in our view) lower and upper bound for the part of companies that could be considered 'sinful' in some way. Firstly, we point to data from Trinks and Scholtens (2017), who estimate that companies dealing with the fourteen controversial issues they study constitute 10% of the number of S&P 500 stocks, and 12% of the market capitalization of companies from that index. But this is just a lower bound, given the growing number of issues that could contribute to the company being treated as 'sinful'. Secondly, we point the reader to a US survey consisting of over 14,500 employees, in which 23% of them indicated that (Ivcevic et al., 2020). Lastly, we suggest that ESG ratings could be used as proxies for being sinful (e.g., if a company is classified as a 'laggard' by MSCI).

We also provide estimates for the premium resulting from working for morally controversial companies, that are provided Schneider et al. (2020). In that study, it was estimated that tobacco companies pay a 35% higher wage than neutral (conventional) companies. Given that tobacco companies are (probably) one of the most controversial ones, this (probably) serves as an upper bound for the effect (which we acknowledge in the text). This should be a useful proxy for the reader, nonetheless.

5. In the discussion section, the authors argue that their results suggest that men might internalize the preferences of their daughters. I find this result quite surprising. Does this mean the daughters are already grown-up individuals? If so, it would be useful to bring up some evidence on the demographics of these daughters/sons. My interpretation would be that having a daughter may carry a different emotional load and men may want to cater to this social norm pressure. Can the authors rule out such explanation? What is the basis for such inference?

This is a great observation, something that indeed requires clearing up. We assume that the Reviewer is referring to the following, indeed misleading sentence from our discussion: "This effect suggests that men might internalize the preferences of their

daughters, making their judgments and behaviors more in line with those manifested by women". In truth, we actually believe in the interpretation provided by the Reviewer, that being a parent to a daughter causes parents to behave differently (due to, e.g., expected or actual social pressures). It should not require parents to observe the behavior of their own child, and instead be the result of a quick adaptation to the reality of being a parent to a daughter. Pogrebna et al. (2018) provide some evidence that this is the case, using data from future parents who have just found out about the sex of their not-yet-born child. Our own results (reported in Table S4) are also generally consistent with this interpretation, suggesting that for the daughter effect to work, it does not require years of socialization (parents "growing up" with their children).

We have now amended the introduction, to make clear that this is our (and the Reviewer's) assumption concerning the mechanism behind potential daughter effects.

Overall, we are indebted to the Reviewer for finding the time to review our manuscript. This is kind. The comments are insightful, and have led to revisions that the readers will benefit from.

We'd also like to take the opportunity to thank the Reviewer for the pioneering work that has inspired not only this paper, but a whole research project that we are involved in. Thank you!

References

Ivcevic, Z., Menges, J. I., & Miller, A. (2020, March 20). How Common Is Unethical Behavior in U.S. Organizations? *Harvard Business Review*. https://hbr.org/2020/03/how-common-is-unethical-behavior-in-u-s-organizations

Johansson-Stenman, O., & Svedsäter, H. (2012). Self-image and valuation of moral goods: Stated versus actual willingness to pay. *Journal of Economic Behavior & Organization*, *84*(3), 879–891. https://doi.org/10.1016/j.jebo.2012.10.006

Pogrebna, G., Oswald, A. J., & Haig, D. (2018). Female babies and risk-aversion: Causal evidence from hospital wards. *Journal of Health Economics*, *58*, 10–17. https://doi.org/10.1016/j.jhealeco.2017.12.006

Schneider, F., Brun, F., & Weber, R. A. (2020). *Sorting and Wage Premiums in Immoral Work* (SSRN Scholarly Paper ID 3646150). Social Science Research Network. https://doi.org/10.2139/ssrn.3646150

Trinks, P. J., & Scholtens, B. (2017). The Opportunity Cost of Negative Screening in Socially Responsible Investing. *Journal of Business Ethics*, *140*(2), 193–208. https://doi.org/10.1007/s10551-015-2684-3
